# Health-related quality of life, handgrip strength and falls during detraining in elderly habitual exercisers

**DOI:** 10.1186/s12955-017-0800-z

**Published:** 2017-11-21

**Authors:** Izaro Esain, Ana Rodriguez-Larrad, Iraia Bidaurrazaga-Letona, Susana María Gil

**Affiliations:** Department of Physiology, Faculty of Medicine and Nursing, University of the Basque Country (UPV/EHU), Barrio Sarriena s/n, E-48940 Leioa, Bizkaia Spain

**Keywords:** Quality of life, Elderly, Supervised physical activity, Fall

## Abstract

**Background:**

The effects of regular exercise on physical functioning and health-related quality of life (HRQOL) have been thoroughly studied. In contrast, little is known about the changes which occur following cessation of activity (detraining). Here, we have investigated the effect of a 3 month detraining period on HRQOL and on handgrip strength in elderly people who had regularly exercised, and examined the association of these variables with falls.

**Methods:**

Thirty-eight women and 11 men (mean age, 75.5±5.7 years) took part in a supervised physical exercise program for 9 months, followed by a 3 month detraining period. Participants completed the SF-36 HRQOL questionnaire at the beginning of detraining (baseline) and 3 months later. Handgrip strength and number of falls were also recorded.

**Results:**

Participants had been exercising for 12.1±8.7 years. After the detraining period, we found a significant (*p* < 0.001–-0.05) decline in all SF-36 dimensions, with the exception of handgrip strength. Women presented a larger decline (*p* < 0.05) in more items than men. During the detraining period, 18.4% participants had a fall incident. HRQOL declined in both fallers and non-fallers during detraining. Interestingly, fallers already had at baseline significantly lower values in physical functioning (*p* < 0.05), emotional role (p < 0.05) and mental health (*p* < 0.01), than non-fallers.

**Conclusions:**

An important decline was found in most items of the SF-36 following a 3 month detraining period, particularly in women. In contrast, strength of the upper limb was not affected by the detraining. The prior lower HRQOL values of those who will subsequently fall suggest that this criterion should be studied as a candidate risk factor for falls. Efforts should be made to encourage the elderly to continue with exercise activities and/or to shorten holiday break periods, in order to maintain their quality of life.

**Trial registration:**

The protocol was registered as a clinical trial in the ANZCTR (trial ID: ACTRN12617000716369).

## Background

In recent years, the proportion of older people is increasing all over the world. According to the latest data from the World Health Organization (WHO), between 2015 and 2050 the proportion of older people will increase from 12 to 22%, which means an increase from 900 million to 2 billion elderly people 60 years old [1]. Aging is considered a deleterious, progressive, intrinsic and universal process which occurs in every living being over time as an expression of the interaction between the individual’s genetic program and its environment [2]. One of the implications of aging is that falls and fall-related injuries are common in older populations. In fact, about one-third of people over the age of 65 fall at least once a year [3]. These falls are a cause of high mortality and morbidity and have negative effects on quality of life and independence [4].

It has been demonstrated that physical activity is an important factor to achieve healthy aging [5]. According to May et al., physical activity provides a longer life in good health [6], and physical training reduces the effects of aging on functional fitness [7]. Among other factors, falls in elderly people are related to low levels of muscular strength [8] and impaired balance [9]; thus, physical activity constitutes and optimal preventive strategy and plays an important role in the prevention of falls [10].

Handgrip strength is an indicator of general muscle strength and it is associated with fragility and propensity to fall [11]. Moreover, the decline in handgrip strength is related to the loss of physical functionality [12]. In a systematic review Bohannon concluded that grip strength should be considered as a useful measure for the screening of the health condition of elderly individual [13]. The utilization of grip strength is supported because of its predictive validity, measurement properties, simplicity, portability and affordability [13].

According to the WHO, quality of life is defined as the individual’s perception of their position in life, in the cultural context, in the value system in which they live and in relation to their goals, expectations, norms and concerns. This concept also encompasses physical health, psychological status, level of independence, social relationships, environmental factors and also personal beliefs [14]. In order to quantify health-related quality of life, the SF-36 health questionnaire has been widely used, being one of the most employed instruments [15, 16], particularly for elderly people [17–19]. The functional autonomy of the elderly affects their quality of life, with exercise being a protector and precursor of this autonomy [20]. Therefore, the promotion of active aging with more years free from functional limitations, coupled with compensatory strategies supporting autonomy and independence among older adults, are fundamental for their quality of life and happiness [21].

While the beneficial effects of regular physical exercise (*training*) on physical functioning and quality of life have been widely reported in the literature [22, 23], less attention has been given to changes occurring following cessation of activity [24], i.e. *detraining*. Most published studies in this regard have analyzed the effect of detraining on muscle function and physical performance [24, 25]. In this sense, it has been observed that following a 9 week multicomponent exercise training program in which elderly subjects improved in strength, agility, flexibility and balance, 6 weeks of detraining was found to induce a significant decline in functional fitness [25]. Similarly, Bocalini et al. observed that functional fitness improved in women older than 62 years who took part in water-based exercises for 12 weeks, three times per week; however, after 6 weeks of detraining they displayed a substantial decline in all the functional fitness tests [26].

Surprisingly, little data is available regarding the influence of detraining on quality of life. In this regard, questionnaires such as the Nottingham Health profile [27], the University of Queensland Quality of Life Questionnaire [24] and WHO quality of life questionnaires [26], have been used, but to the best of our knowledge, only one study has analyzed the effect of the cessation of exercise on the SF-36 in healthy participants [28]. In that study, the authors observed that after a year of training followed by 3 months of detraining, agility/dynamic balance, lower body strength and flexibility were the components most affected, while the HRQOL remained unchanged [28]. Nevertheless, in the mentioned study quality of life was displayed as a unique score; consequently, a more profound analysis of the effects of detraining on the different items of the SF36 would be worthwhile.

The majority of studies in this area have investigated the effect of detraining after a period of training in previously sedentary people. Thus, little is known about the effect of detraining on habitual exercisers. The availability of the older adult to take extended holidays, coupled with volunteering or family commitments [24], and the interruption of exercise classes due to summer vacations may lead to periods of training cessation. In these situations, it is not uncommon that people accustomed to regular exercise, particularly the elder, complain about stiffness of the joints, increased body pain and a decline in their well-being. Taking into account the benefits of physical activity on the quality of life of the elderly, it would be interesting to ascertain to what extent quality of life changes during the detraining period in habitual elderly exercisers. Moreover, it is of interest to study if a period of detraining impairs strength. Thus, it would be interesting to understand if there is a link between detraining and the number of falls in the elderly, and its relationship with quality of life and strength. Thus, we aimed to evaluate the hypothesis that a period of exercise cessation in habitual exercisers would deteriorate their quality of life, particularly in terms of the items related to physical function. Also, due to the fact that detraining produces a decline in physical function, it could be expected that the number of falls would increase in elderly people accustomed to regular exercise, and that this would consequently affect their quality of life.

The purpose of the present study was to evaluate the effect of 3 months of detraining on elderly men and women, who had previously practiced regular supervised physical activity, in terms of quality of life and handgrip strength and also its relationships with falls.

## Methods

### Study design and recruitment

Participants were recruited from a supervised physical exercise program offered to people aged over 65 years in a public sports center in Getxo (Bizkaia, Basque Country). The inclusion criteria were to be enrolled in a supervised group exercise program for people aged over 65 during the previous 9 months, at least. All potential participants received detailed study information in their sports center through the research team. Sixty-eight older adults met the inclusion criteria and were contacted for eligibility; nine of them declined to participate, therefore 57 participants were recruited for the study and completed the baseline assessment. For different reasons eight people left the study so at the end we analysed 49 subjects. Objectives, measurement variables and other details were also explained orally. Informed consent was obtained from each participant who signed it after fully understanding the procedures. The study was approved by the Ethics Committee of the University of the Basque Country (UPV/EHU) (M10_2015–204). The protocol was registered as a clinical trial in the Australian New Zealand Clinical Trials Protocol (trial ID: ACTRN12617000716369).

The participants were attending two group sessions/week multicomponent program. Each session lasted 50 min. The session consisted of a 15 min warm-up to prepare for the session. This consisted of performing joint movements while listening to music; subsequently, they played a simple racquet game using a soft ball. During the main part of the session, they performed strength exercises of major muscle groups, static and dynamic balance exercises, and reaction speed exercises, finishing with stretching. All sessions were guided by the same experienced professional throughout the training period. The program had a length of 9 months and had a scheduled cessation period of 3 months during the summer, for summer holidays. The detraining period was arranged to coincide with this annual break. During detraining, participants were asked to maintain their normal activities of daily living, and to abstain from participating in supervised exercise programs. All the participants were scheduled to stop the exercise program. They were aware of this situation at the beginning of the study.

### Study measures

Measurements were performed twice: First, at the end of the 3 month exercise program, i.e. before discontinuing the program due to the summer period (detraining period), in June. These measurements were considered the baseline measurements. Second, measurements were undertaken again 3 months later, after the 3 months of the detraining period, in September.

The primary outcome measure was the difference in quality of life at the end of the detraining period assessed by scoring each dimension of the HRQOL. This was measured using the 36-item short form survey (SF-36), which is a generic questionnaire whose translation into Spanish has been validated [16]. It is designed to measure the self-reporting health status of the individual. The 36 items are grouped into 8 different dimensions: physical functioning, physical role functioning, bodily pain, general health, vitality, social role functioning, emotional role functioning and mental health. Each subscale score was transformed according to the manual from 0 to 100, with 0 being the worst and 100 the best.

The second outcome was the number of falls during the detraining period, which is defined as an unexpected event in which the participant comes to rest on the ground, floor or lower level [29]. To measure this, participants were asked about any falls which had occurred during the detraining period. Age, gender and anthropometric data were also recorded. Weight and height were measured using Seca (Model 869) instruments; from this, the body mass index was calculated (BMI, kg/m^2^). The circumference of the waist and hip was measured and the waist-hip ratio calculated. All measurements were preformed following the standards of the International Society for the Advancement of Kinanthropometry [30].

Grip strength has been recommended as an assessment technique for the measurement of muscle strength and muscle function in clinical practice [31]. Moreover, lower values of grip strength have been related to an increased number of falls in adults [32]. Therefore, grip strength of the dominant hand was measured using a Jamar Plus digital hand dynamometer. Subjects were seated with back, pelvis, and knees as close to 90 degrees as possible, shoulder adducted and neutrally rotated, elbow flexed at 90 degrees, forearm neutral and wrist held between 0 and 15 degrees of ulnar deviation. The arm was not supported by examiner or armrest and the dynamometer was presented vertically and in line with the forearm [33]. The participants were given verbal encouragement to achieve maximal strength. The mean of three trials was recorded.

### Statistical analysis

Sample size was calculated to detect minimal significant effects on the variable of handgrip strength, accepting a two-sided significance level of 0.05, a clinically relevant improvement of 2 kg in handgrip strength and a standard deviation of 3 kg, and a power of 90%, 47 individuals are required. Presuming a drop-out of 5%, the resultant sample size was determined in 50 individuals.

Data was analyzed using Statistical Package for Social Sciences IBM software (SPSS version 21.0). Normality of data was assessed using the Kolmogorov-Smirnov test. Mean and standard deviations were used as descriptive statistics. To identify significant differences (with respect to baseline) in all variables after the 3 months of detraining (3 month), a Wilcoxon test for dependent samples was used. Also the change of each variable was calculated as a percentage using the following formula: (3 month - baseline)/baseline*100.

We used Mann-Whitney U statistics for 2 group comparisons. To compare the results of the present study to data of the general population [34] the effect size was used, evaluating Cohen’s d parameter. Threshold values for effect size statistics were 0.2, 0.5 and 0.8 for small, medium and large effect sizes, respectively [35]. Mean differences between baseline and 3 months were calculated, as well as 95% confidence intervals. In all cases, the level of significance was set at *p* < 0.05.

## Results

As can be observed in Table 1, no significant changes were observed in the anthropometric measurements after the detraining period.

**Table 1:** Characteristics of the study sample

	Baseline m±sd	3 months m±sd
Age (years old)	75.55 ± 5.77	–
Female	38 (77.6%)	–
Male	11 (22.4%)	–
Height (cm)	159.80 ± 7.34	159.81 ± 7.34
Weight (kg)	72.63 ± 12.98	72.63 ± 13.02
BMI	28.41 ± 4.58	28.40 ± 4.56
Waist-Hip ratio	0.90 ± 0.06	0.92 ± 0.06
Years of physical activity	12.10 ± 8.80	–
Non-fallers (number)	–	40 (81.6%)
Fallers (number)	–	9 (18.4%)

*Abbreviations* **:** *BMI* body mass index, *M* mean, *SD* standard deviation

Regarding the HRQOL, statistically significant (*p* < 0.05-*p* < 0.001) differences were observed in all the items after the detraining period; see Table 2.

**Table 2:** Health-related quality of life (HRQOL) and handgrip strength of participants before (baseline) and after (3 months) detraining

	Baseline m±sd	3 months m±sd	d	Mean difference (95% CI)	*p*-value
HRQOL (SF-36) Physical func.	91.4±8.9	84.1±11.9	−0.61	−7.3 (−9.5-(−)5.2)	<0.001
Physical role func.	96.4±12.5	87.8±28.9	−0.29	−8.7 (−17.7–0.3)	0.05
Bodily pain	80.8±19.9	66.0±22.1	−0.66	−14.7 (−20.6-(−)8.8)	<0.001
General health	71.6±17.8	67.6±17.1	−0.23	−3.9 (−7.7-(−)0.2)	0.02
Vitality	77.4±15.4	69.2±15.1	−0.54	−8.2 (−11.7-(−)4.7)	<0.001
Social role func.	94.6±9.5	85.9±18.2	−0.48	−8.8 (−13.8-(−)3.7)	0.001
Emotional role func.	91.2±25.3	77.5±36.9	−0.37	−13.7 (−23.3-(−)4.1)	0.004
Mental health	77.7±17.5	71.4±16.2	−0.39	−6.4 (−10.8-(−)1.9)	<0.001
STRENGTH					
Handgrip strength (kg)	25.2±6.4	25.3±6.4	0.01	0.12 (−0.63–0.84)	>0.05

*Abbreviations* **:** *Func.* functioning, *HRQOL* health related quality of life, *CI* confidence interval, *m* mean, *sd* standard deviation, *d* Cohen’s d

When we analyzed the HRQOL taking into account the sex of the participants, there were no statistically significant differences between men and women. However, at baseline, men displayed higher values than women in all items. The exception was social functioning in which women displayed slightly higher values than men (95.0 ± 9.5 vs. 93.1 ± 8.5) (Table 3).

**Table 3:** Health-related quality of life (SF-36) and handgrip strength of participants before (Baseline) and after (3 months) the detraining period, by sex

		Female (m±sd)	Male (m±sd)
HRQOL (SF-36) Physical func.	Baseline	90.2 ± 9.6	95.4 ± 3.5
	3 months	83.0 ± 12.6^***^	87.7 ± 9.04^*^
Mean difference (95% CI)		−7.2 (−9.7-(−)4.7)	−7.7 (−13.2-(−)2.2)
Physical role func.	Baseline	96.0 ± 12.6	97.7 ± 7.5
	3 months	88.1 ± 28.3	86.3 ± 32.3
Mean difference (95% CI)		−7.8 (−18.0–2.2)	−11.3 (−34.3–11.6)
Bodily pain	Baseline	79.3 ± 20.4	85.5 ± 17.7
	3 months	64.4 ± 23.0^***^	71.4 ± 18.3^*^
Mean difference (95% CI)		−14.8 (−21.9-(−)7.8)	−14.0 (−26.1-(−)2.0)
General health	Baseline	70.0 ± 18.8	76.9 ± 12.6
	3 months	67.3 ± 18.25	68.2 ± 13.2
Mean difference (95% CI)		−2.6 (−6.4–1.2)	−8.6 (−20.0–2.7)
Vitality	Baseline	77.3 ± 16.2	77.2 ± 12.5
	3 months	68.9 ± 16.4^***^	70.0 ± 10.0
Mean difference (95% CI)		−8.4 (−12.4-(−)4.4)	−7.2 (−15.8–1.2)
Social role func.	Baseline	95.0 ± 9.8	93.1 ± 8.5
	3 months	87.0 ± 18.2^**^	81.6 ± 17.9
Mean difference (95% CI)		−7.9 (−13.5-(−)2.4)	−11.5 (−25.2–2.1)
Emotional role func.	Baseline	89.4 ± 28.0	96.9 ± 10.0
	3 months	79.7 ± 36.8^*^	69.5 ± 37.9^*^
Mean difference (95% CI)		−9.7 (−20.4–1.0)	−27.4 (−49.5-(−)5.3)
Mental health	Baseline	76.0 ± 18.3	83.6 ± 12.9
	3 months	71.0 ± 17.2^**^	72.3 ± 12.5^*^
Mean difference (95% CI)		−4.9 (−10.2–0.3)	−11.2 (−19.4-(−)3.05)
STRENGTH			
Handgrip strength (kg)	Baseline	22.6 ± 3.6	34.3 ± 6.1^‡‡‡^
	3 months	22.8 ± 3.5	34.1 ± 7.1^‡‡‡^
Mean difference (95% CI)		0.2 (−0.5–0.9)	−0.2 (−2.9–2.5)

*Abbreviations: HRQOL* health related quality of life, *CI* confidence interval, *Func.* functioning

Notes**:** ^*^p < 0.05, ^**^p < 0.01, ^***^p < 0.001, statistically significant differences, baseline vs. 3 months

^‡‡‡^ *p* < 0.001, statistically significant differences female vs. male

After the detraining period, both women and men displayed statistically lower values (*p* < 0.05-*p* < 0.001) in the dimensions of physical functioning, bodily pain, emotional role functioning and mental health. Additionally, women had lower values in the dimensions of vitality (p < 0.001) and social functioning (*p* < 0.01).

During the three-month period of study, 9 (18%) falls were reported. Of these, 8 (89%) were women and 1 (11%) men. At baseline, participants who afterwards had a fall, displayed statistically lower values in physical functioning (*p* < 0.05), emotional role functioning (*p* < 0.05) and mental health (*p* < 0.01) than the non-fallers. Moreover, after the detraining period, fallers had lower values in physical role functioning (*p* < 0.05), general health (*p* < 0.05) and mental health (*p* < 0.05) (Table 4).

**Table 4:** Health-related quality of life (SF-36) and handgrip strength before (Baseline) and after (3 months) the detraining period in fallers and non-fallers

		Non-Fallers (m±sd)	Fallers (m±sd)
HRQOL (SF-36) Physical func.	Baseline	91.6 ± 9.4	90.5 ± 5.8^‡^
	3 months	84.7 ± 12.7^***^	81.1 ± 7.8^*^
Mean difference (95% CI)		−6.8 (−9.4-(−)4.3)	−9.4 (−13.9-(−)4.9)
Mean change (%)		−7.6 ± 9.0	−10.4 ± 6.4
Physical role func.	Baseline	96.2 ± 13.3	97.2 ± 8.3
	3 months	92.5 ± 22.0	66.6 ± 45.0^‡^
Mean difference (95% CI)		−3.7 (−12.3–4.8)	−30.5 (−62.0–0.9)
Mean change (%)		−2.5 ± 54.1	−33.3 ± 45.0
Bodily pain	Baseline	81.1 ± 19.9	78.8 ± 20.7
	3 months	68.3 ± 20.7^**^	56.0 ± 26.1^*^
Mean difference (95% CI)		−12.8 (−19.5-(−)6.1)	−22.8 (−35.9-(−)9.8)
Mean change (%)		−13.4 ± 25.3	−30.4 ± 25.6
General health	Baseline	73.9 ± 16.9	60.7 ± 18.5
	3 months	70.6 ± 15.3	54.2 ± 19.1^‡^
Mean difference (95% CI)		−3.3 (−7.4–0.7)	−6.5 (−17.6–4.5)
Mean change (%)		−1.7 ± 21.4	−9.1 ± 22.8
Vitality	Baseline	78.6 ± 13.0	71.6 ± 23.3
	3 months	71.5 ± 12.5^**^	58.8 ± 21.4^*^
Mean difference (95% CI)		−7.1 (−11.1-(−)3.1)	−12.7 (−19.9-(−)5.5)
Mean change (%)		−7.4 ± 16.9	−19.8 ± 15.8
Social role func.	Baseline	95.6 ± 8.27	90.2 ± 13.6
	3 months	88.6 ± 15.7^**^	73.5 ± 23.7^*^
Mean difference (95% CI)		−7.0 (−12.5-(−)1.4)	−16.7 (−30.3-(−)3.1)
Mean change (%)		−6.6 ± 18.3	−19.2 ± 18.9
Emotional role func.	Baseline	94.1 ± 21.2	77.7 ± 37.7^‡^
	3 months	82.4 ± 31.2^*^	55.5 ± 52.7
Mean difference (95% CI)		−11.7 (−22.1-(−)1.3)	−22.2 (−50.8–6.4)
Mean change (%)		−9.5 ± 45.3	−37.5 ± 51.7
Mental health	Baseline	80.9 ± 16.1	63.5 ± 16.7^‡‡^
	3 months	73.9 ± 15.4^***^	60.0 ± 15.4^‡^
Mean difference (95% CI)		−7.0 (−12.3-(−)1.7)	−3.5 (−10.8–3.7)
Mean change (%)		−2.9 ± 48.6	−4.6 ± 14.3
STRENGTH			
Handgrip (kg)	Baseline	25.2 ± 7.0	25.3 ± 3.6
	3 months	25.4 ± 7.1	25.0 ± 2.5
Mean difference (95% CI)		0.2 (−0.7–1.1)	−0.3 (−1.9–1.3)
Mean change (%)		1.3 ± 10.0	−0.5 ± 7.7

*Abbreviations*: *HRQOL* health-related quality of life, *CI* confidence interval, *Func.* functioning

Notes:^*^ *p* < 0.05, ^**^ *p* < 0.01, ^***^ *p* < 0.001, statistically significant differences baseline vs. 3 months

^‡^p < 0.05, ^‡‡^p < 0.01, statistically significant differences non-fallers vs. fallers

When we compared the values at baseline and after 3 months, we observed that both fallers and non-fallers had lower values in all dimensions after the detraining period. In the non-fallers group, we observed statistically significant lower values in the dimensions of physical functioning (*p* < 0.001), bodily pain (*p* < 0.01), vitality (*p* < 0.01), emotional role functioning (*p* < 0.05) and mental health (*p* < 0.001). In contrast, in the group that suffered a fall, statistically significant differences (*p* < 0.05) were found in the dimensions of physical functioning, bodily pain, vitality and social role functioning.

Regarding the handgrip strength, men displayed larger values than women (*p* < 0.001) (Table 3). No changes were observed along the detraining period (Table 2). In addition, handgrip strength was similar in fallers and non-fallers both at the beginning and the end of the study (Table 4).

## Discussion

The benefits of exercise and physical activity on the different aspects of health, including quality of life, have been widely investigated; however, little is known about the impact of the cessation of supervised physical exercise on the quality of life of healthy elderly people who exercise regularly. Taking into account that supervised exercise programs may have predicted (summer holidays) or unpredicted (illnesses or family care) cessation periods, the aim of the present study was to ascertain the effect of a detraining period on the quality of life and handgrip strength, and the relation to falls in elderly exercisers. An important decline was found in most items of the SF-36 following a 3 month detraining period, particularly in women and in those participants who had a fall. Moreover, fallers already had at baseline significantly lower values in several items related to the quality of life prior to the fall incident.

It is well recognized that physical activity is positively related to the quality of life of elderly people [23, 36]. This notion, coincides with the fact that participants of the present study, who had been attending exercise classes for an average of 12 years, displayed larger values in most items of the quality of life than people of the general population [34] reinforcing the positive effect of physical exercise on the quality of life of the aging adults.

Interestingly, this better quality of life of the habitual exercisers of the present study suffered an important decline only after 3 months of exercise cessation. Only a few studies have analyzed the effect of detraining on quality of life, delivering conflicting results. While some authors did not observe any changes in quality of life after a 3 month detraining period [28, 37], Bocalini et al. observed a significant decrement at 4 weeks and a larger one at 6 weeks, comparable to the present results [26]. The cessation of physical exercise has been associated with reduced aerobic capacity, body strength, agility, flexibility and static balance [26] which may explain the decline in the items related with physical function (physical functioning, role physical, bodily pain and general health). Moreover, since participants in the present study had been exercising in groups for a long time, it is possible that those who lived alone or who looked after a husband/spouse or other family member during the summer break would, as a consequence of detraining, have had fewer opportunities for socialization and breaking their routines, thereby leading to a decline in the psycho- and social related items of the quality of life.

It has previously been demonstrated that women in the general population have a lower quality of life than men [38, 39]. Although not statistically significant, the women in the present study also reported lower values in most items of the HRQOL compared to men, at baseline. Nevertheless, it is remarkable that the effect of detraining was larger in women than in men. In fact, whereas both men and women experienced a decline in physical functioning, bodily pain, emotional role functioning and mental health, women declined additionally in vitality and social role functioning. Consequently, in order to maintain their quality of life it is advisable to encourage people who attend guided exercise programs, to engage in some kind of group exercise or other type of gathering in order to maintain their well-being during long periods of cessation of physical activity. This advice should particularly be delivered to women exercisers.

During the 3 month detraining period, 18.4% of participants suffered a fall, which is higher than what could be expected [40]. Toulette et al. observed that the beneficial effects of training significantly dropped in both fallers and non-fallers during 3 months of detraining, particularly in terms of walking parameters (walking speed, cadence and stride length) and of balance [41]. We could not observe any changes in the handgrip strength during the detraining and there were not differences between fallers and non-fallers. Nonetheless, it is likely that detraining leads to deterioration in strength of the lower limb, coordination and balance, increasing the risk of falls, which should be counterbalanced by different activities in the periods of cessation of the supervised program.

It is remarkable that even before the detraining period, those participants who had a fall already displayed lower values in all the HRQOL items, with differences being significant in physical role functioning, emotional role functioning and mental health compared to the non-fallers. Research about the relationship between the quality of life preceding a fall is scarce but interesting. It has been observed that the lowest quality of life scores was associated with a greater risk of falls [42]. Moreover, patients who suffered a wrist [43] and a hip [44] fracture following a fall, retrospectively recalled lower “pre-injury” values in various items of the SF-36, than non-fallers. Hence, the observed lower quality of life of fallers may be an interesting sign which may merit further research.

Moreover, participants who had a fall after the detraining period scored lower absolute HRQOL values and exhibited a larger decline than non-fallers in all dimensions, with differences in physical role functioning, general health and mental health items being statistically significant. The negative effect of a fall on the quality of life of the elderly has previously been demonstrated [10, 29]. Those people that suffered a fall probably had fear of falling again and it has an important impact on their quality of life [45]. Nevertheless, even after adjusting for the effect of fear of falling [29], the quality of life of fallers remained lower compared to non-fallers. A fall can imply an injury, limitations in physical activity, loss of independence and a decrease in psychological functioning [46], thus having a large impact on quality of life.

## Limitations

The potential limitations of the present study should be taken into account. On the one hand, the study was undertaken in the absence of a control group, the inclusion of which would have allowed comparative data from a group of non-exercising elderly. On the other hand, the present study does not include a very large number of participants. However, it should be borne in mind that the recruitment of elderly people who had continuously been exercising for a long period of time (even years) in order to investigate the effect of detraining on habitual exercisers, is not an easy task. The interesting results obtained in the present study should encourage the design of larger research studies including different groups of participants and different variables.

## Conclusion

There is an important decline in most items of the SF-36 following a 3 month detraining period. This decline is particularly evident in women and it is not related to a loss of handgrip strength. Therefore efforts should be made to either maintain the same type of exercise and/or shorten vacations or holiday break periods, particularly in the case of women. In addition, people who suffer a fall during the detraining period displayed lower HRQOL values not only after but also before the event, thus meriting further attention.

## Abbreviations

BMI: Body mass index
HRQOL: Health-related quality of life
WHO: World Health Organization
